# Case Report: A rare case of hemorrhagic cystic IPAS masquerading as pancreatic neoplasm

**DOI:** 10.3389/fonc.2025.1626836

**Published:** 2025-07-11

**Authors:** Chong-yuan Chen, Yu Yang, Ming-zheng Hu, Rong-chun Xing

**Affiliations:** ^1^ The First College of Clinical Medical Science, China Three Gorges University, Hubei Yichang, China; ^2^ Department of Hepatobiliary Surgery, Yichang Central People’s Hospital, Hubei, China

**Keywords:** Intrapancreatic accessory spleen, hemorrhagic cystic degeneration, pancreatic neoplasm, mucinous cystic neoplasm, differential diagnosis, laparoscopic distal pancreatectomy

## Abstract

Intrapancreatic accessory spleen (IPAS) is a rare congenital developmental anomaly that is typically benign and does not require specific treatment. However, when IPAS undergoes hemorrhage and subsequent cystic degeneration, its clinical symptoms and laboratory findings often lack specificity, and imaging studies can be misleading, frequently resulting in misdiagnosis as pancreatic tumors or other lesions. This poses significant challenges for preoperative diagnosis. We report a case of a 53-year-old woman presenting with vague upper abdominal pain. Preoperative imaging strongly suggested a mucinous cystic neoplasm of the pancreas. However, postoperative pathology following laparoscopic distal pancreatectomy combined with splenectomy confirmed hemorrhagic cystic degeneration of an IPAS. This case highlights the importance of considering hemorrhagic cystic IPAS in the differential diagnosis of pancreatic space-occupying lesions, particularly when imaging findings are atypical or inconclusive. Endoscopic ultrasound-guided fine-needle aspiration (EUS-FNA) is a safe and effective method for obtaining a pathological diagnosis. Enhanced awareness of this condition, combined with meticulous radiological evaluation and necessary histopathological biopsy, can reduce misdiagnosis, avoid unnecessary surgical trauma, and improve individualized treatment strategies.

## 1 Introduction

An accessory spleen (AS) is a relatively common congenital anomaly of splenic development, referring to ectopic splenic tissue that is histologically and functionally similar to the normal spleen (1). Its incidence in autopsy series ranges from 10% to 30% (2). AS arises due to incomplete fusion of splenic primordia during embryogenesis and is typically located near the splenic hilum, the tail of the pancreas, or the greater omentum (1). An intrapancreatic accessory spleen (IPAS) refers to ectopic splenic tissue that is partially or entirely embedded within the pancreatic parenchyma and is considerably rarer than accessory spleens in other locations, thus regarded as an uncommon clinical entity (3).

IPAS is usually asymptomatic and is most often discovered incidentally during imaging studies performed for unrelated reasons (4). On imaging, it generally appears as a well-defined, solid lesion. However, when it lacks the typical “spleen-like” enhancement pattern on contrast imaging, it is easily mistaken for pancreatic neuroendocrine tumors, solid pseudopapillary neoplasms, or metastatic carcinoma, leading to unnecessary anxiety and surgical interventions (5). The preoperative diagnostic rate of IPAS remains low, with most cases confirmed only by postoperative histopathology.

What makes this case unique is the spontaneous hemorrhage within the IPAS, followed by cystic degeneration—an exceedingly rare occurrence (6). The imaging features of cystic IPAS are often more complex and atypical, which increases the risk of misdiagnosis as cystic pancreatic neoplasms, such as mucinous cystic neoplasm as in this case (7). The likely mechanism involves rupture of internal blood vessels within the accessory spleen, followed by hematoma formation, incomplete liquefaction, and eventual fibrous encapsulation (8). This complex pathophysiological process results in non-specific findings on CT and MRI, making it challenging to differentiate from primary cystic neoplasms of the pancreas or hemorrhagic lesions (9).

Therefore, this report aims to present a case of hemorrhagic cystic IPAS confirmed by surgery and histopathology, supplemented by a review of the literature (10). We discuss its clinicopathological features, imaging findings, and diagnostic challenges to improve awareness among clinicians, reduce the rate of misdiagnosis, and guide optimal diagnostic and therapeutic approaches with minimal invasiveness and maximal benefit to the patient (11).

## 2 Case report

A 53-year-old Han Chinese woman was referred to our hospital on August 28, 2024, for “upper abdominal pain lasting one day.” The pain had no clear trigger, was unrelated to eating, did not improve with rest, and was not accompanied by radiating pain to the back. The patient had no significant past medical history or comorbidities, the patient denies any other family history or history of hereditary diseases.

On physical examination, she was alert and oriented, with no scleral icterus. The abdomen was flat and soft, with no distention of superficial abdominal veins, no tenderness, rebound pain, or palpable masses. No hepatosplenomegaly was noted. Murphy’s sign was negative. There was no percussion pain over the liver or kidneys, no shifting dullness, and no lower limb edema. An abdominal CT from an outside hospital revealed a space-occupying lesion in the pancreas.

**Figure 1:**
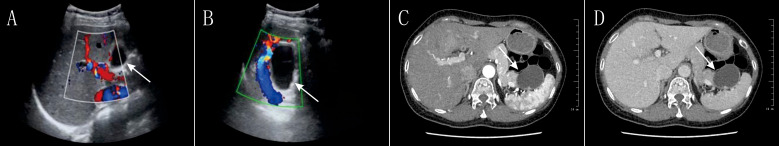
Preoperative Imaging of the Pancreatic Lesion (Ultrasound and CT)Color Doppler Ultrasound Images: **(A, B)** The images show a predominantly cystic, relatively well-defined space-occupying lesion in the region of the pancreatic tail. The Doppler signals (red and blue) demonstrate the vascular distribution around the lesion. These findings have some value in the preoperative differentiation between benign and malignant lesions but are non-specific. Axial Contrast-Enhanced Abdominal CT Images: **(C, D)** The images clearly demonstrate a roundish, low-density cystic lesion located in the tail of the pancreas, near the spleen. Mild enhancement of the cyst wall is visible post-contrast. These CT findings were a key basis for the preoperative suspicion of a pancreatic cystic neoplasm (particularly a mucinous cystic neoplasm).

**Figure 2:**
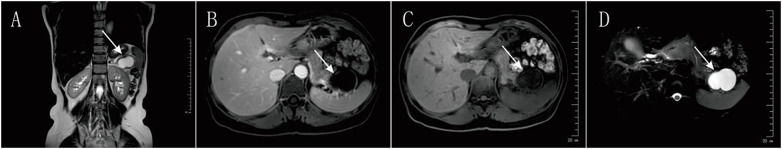
Preoperative Magnetic Resonance Imaging (MRI) of the Pancreatic LesionCoronal T2-Weighted Image: **(A)** This image displays the size of the cystic lesion (high white signal) and its positional relationship to surrounding organs, such as the left kidney. Axial T1-Weighted Images: **(B, C)** These images show the signal characteristics of the lesion on the T1 sequence, which is typically used to assess solid components and internal hemorrhage or proteinaceous content. The literature indicates that while an uncomplicated IPAS should have a “spleen-like” enhancement pattern, these typical features disappear in the presence of hemorrhage and necrosis.Axial T2-Weighted Fat-Suppressed Image: **(D)** On this sequence, the lesion demonstrates a markedly bright, homogeneous high signal, strongly confirming its predominantly fluid-filled nature. This imaging feature is highly similar to that of a mucinous cystic neoplasm (MCN) and was a major contributing factor to the preoperative misdiagnosis.

Initial laboratory tests revealed an elevated white blood cell count (WBC: 12.95 × 10^9^/L), neutrophils (11.46 × 10^9^/L), carcinoembryonic antigen (CEA: 6.9 ng/mL), carbohydrate antigen 19-9 (CA19-9: 102 U/mL), interleukin-2 (IL-2: 6.52 pg/mL), and interleukin-6 (IL-6: 7.4 pg/mL). Aspartate aminotransferase (AST) was 51 U/L, pancreatic amylase was 71 U/L, and lipase was 76 U/L. Other indices, such as hemoglobin, red blood cell count, platelet count, coagulation profile, and alpha-fetoprotein (AFP), were within normal limits.On August 29, 2024, a series of imaging and laboratory tests revealed a cystic mass in the pancreatic tail (**Figure 1**), highly suspicious for mucinous cystic neoplasm of the pancreas (**Figure 2**).

**Figure 3:**
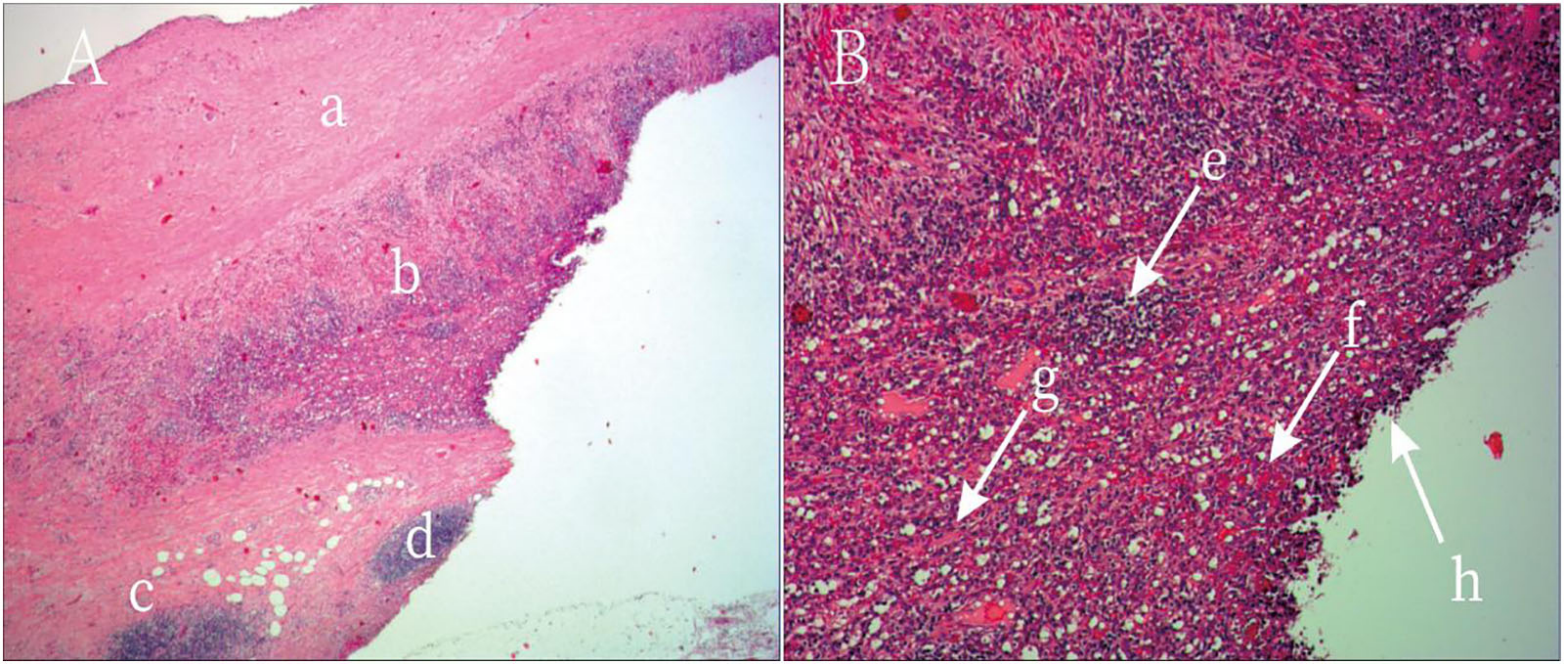
Postoperative Histopathology of the Resected Specimen. Hematoxylin and Eosin (H&E) Stain, Low Magnification: **(A)** This image shows the overall architecture of the lesion. A fibrotic cyst wall is visible superiorly, with dense cellular tissue inferiorly. This clearly displays the transition from the cystic to the solid component of the lesion. **(B)** This is the key image for diagnosis. Under high power, typical splenic tissue architecture can be identified, including lymphocyte-rich white pulp and blood-filled red pulp. Numerous red blood cells are scattered throughout the tissue, confirming extensive hemorrhage. These features collectively constitute the final pathological diagnosis of hemorrhagic cystic degeneration of an intrapancreatic accessory spleen, thereby ruling out a pancreatic neoplasm.

After multidisciplinary discussion, surgical treatment was considered appropriate. Following thorough communication with the patient and her family, written informed consent was obtained. The patient underwent laparoscopic distal pancreatectomy with splenectomy under general anesthesia. The procedure lasted 3 hours and 11 minutes. After induction of anesthesia, the patient was placed in the supine position. The abdomen was prepped and draped in a sterile fashion. A 1 cm infraumbilical incision was made for pneumoperitoneum, and a 10 mm trocar was inserted for laparoscopic access (12, 13). Exploration revealed no abnormalities in the liver, stomach, or intestines (14, 15). The greater omentum was covering the spleen, which was tightly adhered to surrounding tissues (16, 17). After establishing the remaining laparoscopic ports, a 4 × 5 cm cystic mass was identified in the pancreatic tail, closely associated with adjacent vessels (18). The decision was made to perform distal pancreatectomy. The gastrocolic ligament was divided to fully expose the body and tail of the pancreas (15). The anterior surface of the pancreas was incised with an electrocautery knife, and the superior and inferior borders of the pancreatic neck were dissected. The portal vein and superior mesenteric vein were isolated, and the pancreas was transected at the neck. The proximal stump was ligated. Dissection continued along the body and tail of the pancreas, revealing tight adhesion of the mass to splenic vessels. Due to persistent bleeding from splenic vessels, splenic preservation was deemed unfeasible, and a combined splenectomy was performed (19). The splenic vessels were ligated and divided. The pancreatic body and tail, along with the spleen, were resected en bloc following division of surrounding ligaments and short gastric vessels. Hemostasis was achieved, two drains were placed, and the abdominal wall was closed in layers. The resected specimen was reviewed by the patient’s family and sent for pathological examination. The patient was monitored in the intensive care unit for the first 24 hours postoperatively without complications and then transferred to the general ward. Upon regaining consciousness, she reported only mild incisional pain with no other discomfort. One week later, the pathology report confirmed hemorrhagic cystic degeneration of an intrapancreatic accessory spleen located in the pancreatic body and tail (**Figure 3**). The patient was discharged the following day in stable condition.

Final pathology confirmed hemorrhagic cystic degeneration of an accessory spleen in the pancreatic body and tail. Due to limitations in clinical experience, surgical treatment was initiated based on imaging findings alone. Although histopathology is the gold standard for diagnosis in this and many other tumor-related conditions, it is also highly invasive. When imaging and pathology results differ, priority should be given to pathological confirmation. Endoscopic ultrasound-guided fine-needle aspiration (EUS-FNA) or contrast-enhanced ultrasound-guided biopsy is a safe and reliable alternative for obtaining diagnostic tissue samples. However, the procedure carries risks, such as pancreatitis, bleeding, and technical challenges, especially when targeting vascular or deep-seated lesions. From the standpoint of patient welfare, minimizing invasiveness while ensuring diagnostic accuracy remains an important clinical goal.

At 1- and 3-month postoperative follow-up visits, the patient reported no recurrence of abdominal pain or other related symptoms, and her quality of life was comparable to baseline. The patient believed that the timely surgical intervention led to the improvement of her condition and did not consider the decision to undergo surgery before a definitive cause was established to be incorrect.

## 3 Discussion

Intrapancreatic accessory spleen (IPAS) is a rare congenital anatomical variant that originates from incomplete migration and fusion of splenic primordia during embryogenesis, resulting in ectopic splenic tissue embedded within the pancreatic parenchyma (20, 21). Although IPAS shares the same histological structure and immunological function as the normal spleen, its clinical significance lies in the potential for misdiagnosis as pancreatic tumors, particularly pancreatic neuroendocrine tumors (PNETs) or other solid lesions (**Table 1**), based on imaging features (22). Uncomplicated IPAS is typically asymptomatic, often discovered incidentally, and exhibits slow growth, generally requiring no intervention (23). However, when complications arise—such as torsion, infarction, spontaneous rupture with hemorrhage, or, as in this case, hemorrhagic cystic degeneration—patients may present with clinical symptoms, and diagnosis becomes more challenging (24). While IPAS is being increasingly recognized in clinical practice, cystic degeneration—especially hemorrhagic cystic degeneration—remains exceedingly rare (16). On CT or MRI, most IPAS lesions appear as well-defined, rounded masses. In uncomplicated cases, their enhancement pattern often resembles that of the spleen: heterogeneous “mottled” enhancement in the arterial phase, becoming more homogeneous in the portal venous and delayed phases (25). However, when complicated by hemorrhage and necrosis, these lesions may develop cystic changes with heterogeneous internal density or signal, including areas of liquefaction and necrosis (20). The cyst wall may enhance post-contrast, mimicking features of pancreatic cystic neoplasms or pseudocysts. In this case, preoperative imaging demonstrated a cystic lesion in the pancreatic tail, and tumor markers—CA19–9 and CEA—were mildly elevated, leading to an initial diagnosis of mucinous cystic neoplasm. According to previous studies, tumor markers are generally not elevated in cases of IPAS (16). However, concurrent inflammation or tissue necrosis may cause nonspecific elevations, which likely contributed to the misdiagnosis in this patient. Nuclear medicine techniques such as technetium-99m-labeled heat-denatured red blood cell (99mTc-HDRBC) scintigraphy or sulfur colloid liver-spleen scanning are reported to have high diagnostic specificity for IPAS, as these tracers are selectively taken up by splenic tissue. However, these tests are not routinely used in clinical settings. Contrast-enhanced ultrasound (CEUS), capable of real-time evaluation of microvascular perfusion, may demonstrate the characteristic “spleen-like” perfusion pattern of IPAS—heterogeneous hyperenhancement during the arterial phase followed by rapid homogeneous filling (16). CEUS is particularly useful for differentiating IPAS from hypervascular pancreatic neoplasms, such as PNETs. EUS-FNA is considered the gold standard for obtaining a preoperative pathological diagnosis of IPAS, offering direct cytological or histological evidence (5, 26). However, the procedure carries risks, such as pancreatitis, bleeding, and technical challenges, especially when targeting vascular or deep-seated lesions (27, 28). Therefore, safety and feasibility must be carefully assessed before performing EUS-FNA.

**Table 1:** Cross-verification between IPAS, MCN and SCA.

Feature	Hemorrhagic Cystic Intrapancreatic Accessory Spleen (IPAS)	Mucinous Cystic Neoplasm (MCN)	Serous Cystadenoma (SCA)
Etiology	Congenital ectopic splenic tissue with secondary hemorrhage and cystic changes.	Neoplastic proliferation of mucin-producing epithelial cells with an ovarian-type stroma.	Benign neoplastic proliferation of glycogen-rich epithelial cells.
Typical Location	Most commonly in the tail of the pancreas.	Almost exclusively in the body or tail of the pancreas.	Can occur anywhere in the pancreas, but slightly more common in the body or tail.
Clinical Presentation	Often asymptomatic unless complications like hemorrhage occur, which can cause vague abdominal pain.	Often asymptomatic and found incidentally. Can cause non-specific abdominal pain.	Usually asymptomatic and discovered incidentally.
CT Imaging	Appears as a well-defined cystic lesion. Post-contrast imaging may show enhancement of the cyst wall. When uncomplicated, it shows “spleen-like” enhancement (heterogeneous in the arterial phase, homogenous in later phases). Hemorrhage can create complex internal features.	Typically a unilocular or multilocular macrocystic lesion. Peripheral, “eggshell” calcification can be present. Does not communicate with the pancreatic duct.	Classically a “honeycomb” pattern of multiple small microcysts, though macrocystic variants exist. A central scar with “sunburst” calcification is pathognomonic but not always present.
MRI Imaging	Similar to CT, showing a complex cystic mass. The signal characteristics can vary depending on the age of the hemorrhage.	High signal intensity on T2-weighted images due to mucin content.	The microcysts have high signal intensity on T2-weighted images.
Endoscopic Ultrasound (EUS) & FNA	EUS can visualize the lesion, and EUS-guided fine-needle aspiration (FNA) is a key tool for a definitive preoperative diagnosis by obtaining cytological or histological evidence.	EUS can characterize the cyst and FNA can be used to analyze cyst fluid for tumor markers (e.g., CEA) and cytology.	EUS can identify the microcystic nature, and FNA can obtain fluid for analysis.
Treatment	If a definitive preoperative diagnosis is made, less invasive options like local excision or even observation may be considered. In the reported case, a laparoscopic distal pancreatectomy with splenectomy was performed due to the suspicion of a neoplasm.	Surgical resection is generally recommended due to malignant potential.	Often managed with observation for smaller, asymptomatic lesions. Surgery may be considered for larger or symptomatic cysts.

This case presents a detailed clinical and radiological profile of a rare hemorrhagic cystic IPAS. Its major value lies in highlighting the imaging resemblance to pancreatic mucinous cystic neoplasm and sharing the diagnostic pitfalls encountered. Although single-case reports have limited generalizability, they are valuable in raising clinical awareness (17). It is important to include IPAS and its complications (such as hemorrhagic cystic degeneration) in the differential diagnosis of pancreatic cystic or mixed solid-cystic lesions, even when imaging strongly suggests pancreatic neoplasm. Serum tumor markers should not be solely relied upon, as inflammation or necrosis may yield false-positive results. When conventional imaging is inconclusive, a multimodal imaging strategy—including CEUS or nuclear imaging—should be considered. When clinically appropriate and technically feasible, EUS-FNA is a key tool for establishing a definitive preoperative diagnosis and may help avoid unnecessary extensive surgery. Although EUS-FNA (endoscopic ultrasound-guided fine-needle aspiration) is widely recognized as a valuable diagnostic tool for evaluating pancreatic and peripancreatic lesions, it has several limitations. These include the possibility of sampling error due to the heterogeneous nature of the lesion, limited tissue volume for immunohistochemical analysis, and operator dependency. In certain cases, especially when the lesion exhibits extensive cystic degeneration, hemorrhage, or necrosis, the diagnostic yield of EUS-FNA may be significantly reduced. If preoperative diagnosis of hemorrhagic cystic IPAS is achieved, less invasive options such as cyst drainage or local excision may be considered instead of distal pancreatectomy with splenectomy. In this case, although laparoscopic distal pancreatectomy and splenectomy were successfully performed and the patient recovered well, preoperative identification of IPAS might have permitted spleen preservation or a more conservative pancreatic resection, potentially improving long-term quality of life (29).In this case, the laparoscopic distal pancreatectomy with splenectomy was technically successful; it removed the lesion in a minimally invasive manner, led to the patient’s good recovery, and ultimately established a definitive diagnosis through pathology. However, the procedure also highlights the limitations of performing radical surgery when a diagnosis is uncertain. The preoperative misdiagnosis of a benign hemorrhagic cystic accessory spleen as a pancreatic neoplasm led to avoidable “overtreatment”. This approach was not only overly extensive but also sacrificed the spleen, which might have been preserved with a more accurate preoperative diagnosis. Therefore, while the surgery cured the patient, its greater significance is in alerting clinicians to the need to improve preoperative diagnostic accuracy with tools like EUS-FNA to avoid unnecessary surgical trauma.

## 4 Conclusion

Hemorrhagic cystic degeneration of intrapancreatic accessory spleen (IPAS) is a rare condition with nonspecific clinical presentations and laboratory findings. Its imaging characteristics can be highly misleading, often mimicking pancreatic cystic neoplasms, which makes preoperative diagnosis particularly challenging. This case underscores that for pancreatic space-occupying lesions—especially those presenting with cystic or mixed cystic-solid features and diagnostic uncertainty—hemorrhagic cystic IPAS should be included in the differential diagnosis. Histopathological examination remains the gold standard for confirming the diagnosis. However, increased clinical vigilance, integration of multimodal imaging techniques, and, when appropriate, the use of endoscopic ultrasound-guided fine-needle aspiration (EUS-FNA) for preoperative tissue diagnosis are critical. These strategies help reduce the risk of misdiagnosis, avoid unnecessary extensive surgical resections, and guide the selection of minimally invasive yet effective therapeutic options, thereby ensuring the best possible treatment outcome and long-term prognosis for the patient.

## Data Availability

The original contributions presented in the study are included in the article/supplementary material. Further inquiries can be directed to the corresponding author/s.
